# Advances in experimental animal models of hepatocellular carcinoma

**DOI:** 10.1002/cam4.6163

**Published:** 2023-05-29

**Authors:** Jing Li, Xin Wang, Mudan Ren, Shuixiang He, Yan Zhao

**Affiliations:** ^1^ Department of Gastroenterology The First Affiliated Hospital of Xi'an Jiaotong University Xi'an People's Republic of China

**Keywords:** animal model, hepatocellular carcinoma, transgenic

## Abstract

Hepatocellular carcinoma (HCC) is a common malignant tumor with insidious early symptoms, easy metastasis, postoperative recurrence, poor drug efficacy, and a high drug resistance rate when surgery is missed, leading to a low 5‐year survival rate. Research on the pathogenesis and drugs is particularly important for clinical treatment. Animal models are crucial for basic research, which is conducive to studying pathogenesis and drug screening more conveniently and effectively. An appropriate animal model can better reflect disease occurrence and development, and the process of anti‐tumor immune response in the human body. This review summarizes the classification, characteristics, and advances in experimental animal models of HCC to provide a reference for researchers on model selection.

## Graphical abstract

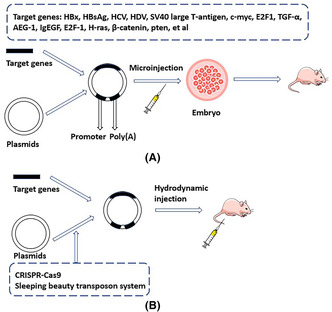

Hepatocellular carcinoma (HCC) is the sixth most common cancer worldwide and has the third highest mortality rate among all tumors.^1^ HCC has many pathogenic factors and multiple pathways involved in its occurrence and metastasis, and drug‐targeted therapy has gradually become a research hotspot in recent years.^2^, ^3^ Animal models are important vehicles for studying the pathogenesis and development of HCC and conducting drug screening.^4^, ^5^, ^6^ In this review, we summarized animal models of HCC and their characteristics to provide not only a reference for researchers on model selection but also a wealth of knowledge for future studies.

Currently, the commonly used animal models of HCC include mice, rats, rabbits, woodchucks, and zebrafish (Figure 1). Mice are the most frequently used animal models because they are highly homologous to humans.^7^ These methods mainly include induced, implanted, and genetically engineered animal models (GEM).^8^, ^9^, ^10^ Rats are genetically similar to humans and can be used as induced and implantation animal models.^11^, ^12^ The rabbit VX2 orthotopic tumor model is used to study quantitative imaging techniques and interventional treatments because of its large size.^13^, ^14^ HCC of woodchuck sharing imaging appearances and biological characteristics with humans is a model for hepatocarcinogenesis induced by the hepatitis B virus (HBV) for preclinical evaluation of the efficacy of treatments based on chemotherapy, gene therapy, and immune therapy.^15^, ^16^ Zebrafish, a valuable non‐mammalian vertebrate, are genetically similar to humans and are mostly used in xenotransplantation and drug screening studies, genetic screening, and monitoring through GEM, such as [Hepatitis B virus X protein (HBx), src] and [HBx, src, p53^−/+^], kras.^17^, ^18^, ^19^, ^20^

**FIGURE 1:**
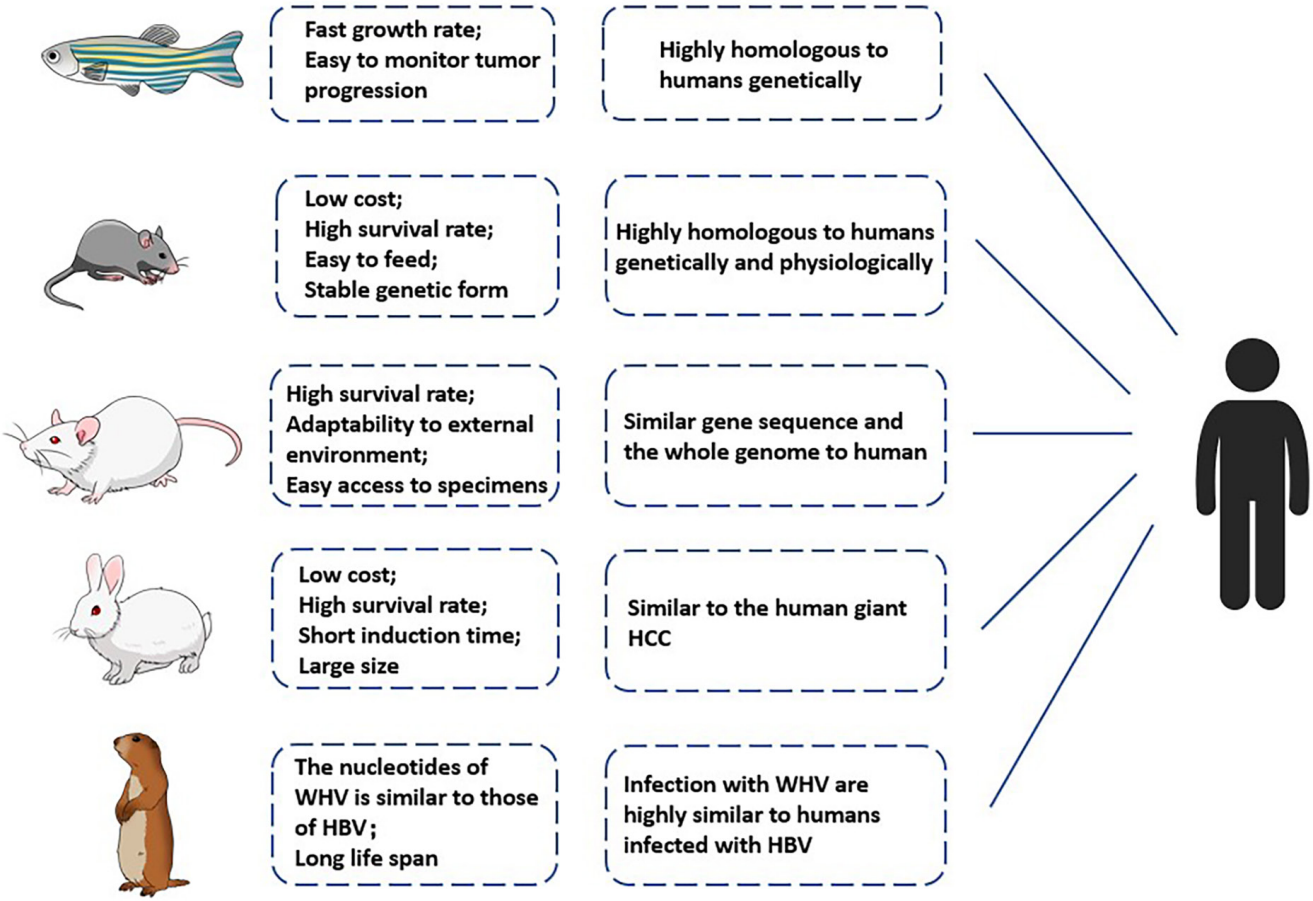
Commonly used animal models of HCC. Animals, including mice, rats, rabbits, woodchucks, and zebrafish with established HCC models, with different advantages and connections with humans. HBV, hepatitis B virus; HCC, Hepatocellular carcinoma.

## 1 INDUCED ANIMAL MODELS

**FIGURE 2:**
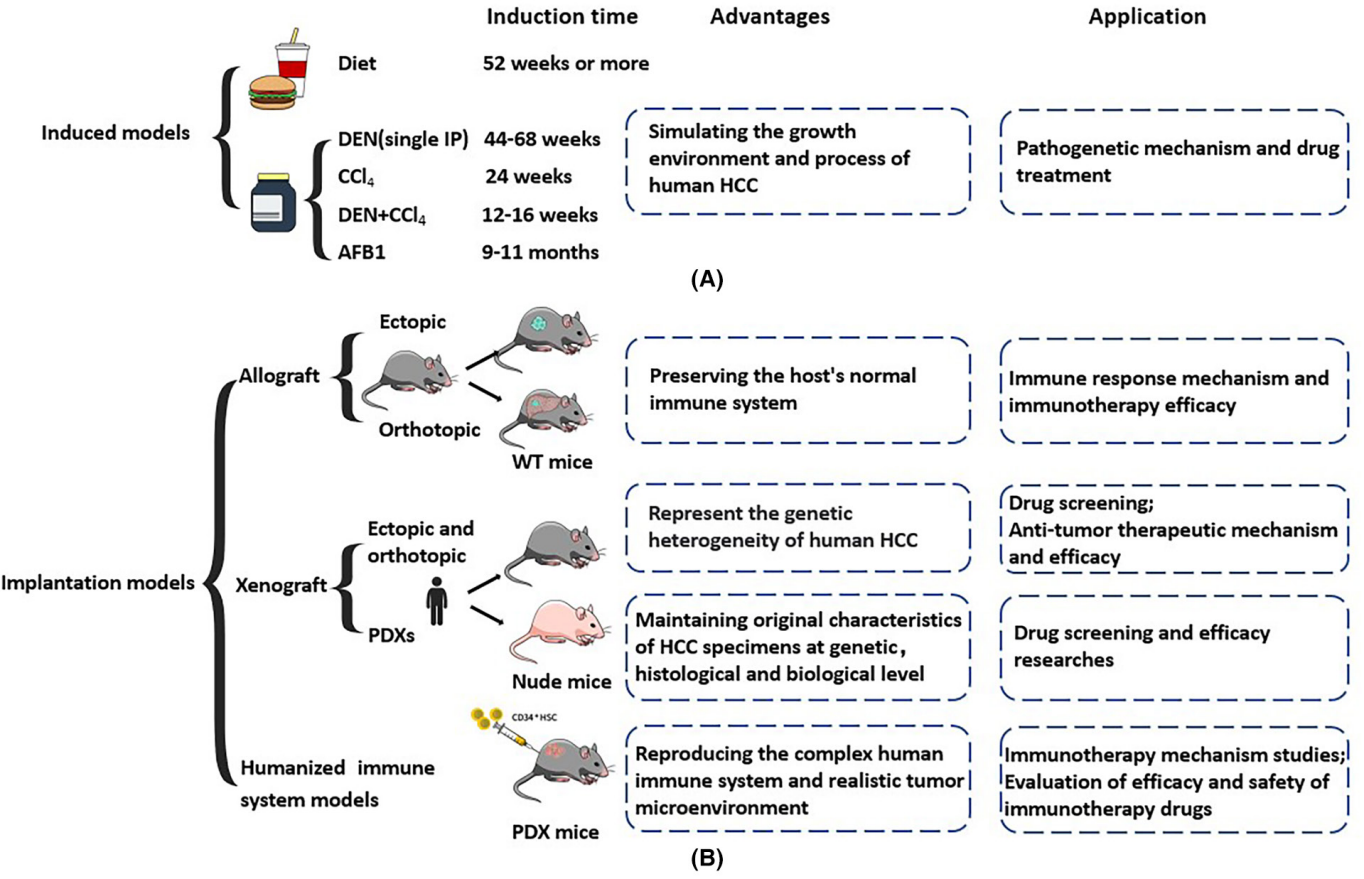
Commonly used induced and implantation models of HCC with different characteristics and clinical applications. (A) Diet and drugs induced models. (B) Implantation models include allograft, xenograft, and humanized immune system models. HCC, Hepatocellular carcinoma; PDXs, patient‐derived xenograft.

Induced models are animals that progress to HCC through long‐term chronic inflammatory reactions in response to artificially intervened cancer‐predisposing factors, including diet, chemical drugs, and biological toxins, which are applied to studies on pathogenetic mechanism and drug treatment (Table 1). In addition, these programs have been used to model liver injury and liver fibrosis.^21^, ^22^ Drugs induce the occurrence of HCC in two ways. First, they can directly cause genetic changes, including interaction with DNA resulting in mutations or damages and chromosome breaks. These drugs include diethylnitrosamine (DEN) and aflatoxin B1 (AFB1). Second, drugs such as carbon tetrachloride (CCl_4_) cause liver damage by destroying cell structures, promoting abnormal cell proliferation, without direct interaction with genetic material (Figure 2).

**TABLE 1:** Induced animal models of hepatocellular carcinoma.

Diet/drugs	Animal	Dose	Route	Mechanism	Induction time	References
CDD	Fischer‐344 rats	‑^a^	Feeding	Epigenetic aberrations of gene expression	52 weeks	25
CDD and amino acid‐defined diet	C57BL/6 mice	‐^a^	Feeding	Temporal changes in microRNA profile	84 weeks	26
CDD + DEN	Sprague–Dawley rats	DEN, 13–15 mg/day	Feeding and drinking	Exacerbation of oxidative injury	16 weeks	27
DEN	C57BL/6 × C3H F1 mice	1.25–5.0 mg/kg	Single IP	Genotoxic hepatocarcinogen	44–68 weeks	37
DEN	Sprague–Dawley rats	30 mg/kg, twice a week for 11 weeks	Long‐term IP	Genotoxic hepatocarcinogen	20 weeks	43
DEN	C57BL/6 mice	0.014% DEN, 6 days per week, with a 1‐day interval on the 7th day for 15 weeks	Oral administration	Genotoxic hepatocarcinogen	13–15 weeks	44
CCl_4_	C57BL/6 mice	0.2 μL (0.32 μg)/g, once a week	Long‐term IP	The formation of reactive oxygen	24 weeks	32
DEN + CCl_4_	C57BL/6 mice	DEN (25 mg/kg or 100 mg/kg), followed by 12 weekly injections of CCl_4_ (0.5 mL/kg)	IP	The formation of reactive oxygen, genotoxic hepatocarcinogen	12–16 weeks	49
AFB1	Gnmt^−/−^ mice and wild‐type C57BL/6 mice	10 mg/kg at 7 days of age, 40 μg at 9 weeks of age	IP	DNA mutation	About 9–11 months and 16–18 months for Gnmt^−/−^ and wild‐type mice, respectively	57
DEN + 2AAF	Wistar albino rats	DEN (150 mg/kg, once a week for 2 weeks), followed by 2‐AAF (20 mg/kg for 3 weeks)	IP for DEN, oral administration for 2‐AAF	Genotoxic hepatocarcinogen	20 weeks	60
Solt–Farber (DEN + partial hepatectomy) + 2‐AFF	Wistar rats	DEN (200 mg/kg), 2‐AFF (10 mg/mL) diet for 3 days	IP for DEN, oral administration for 2‐AAF	Genotoxic hepatocarcinogen	4 weeks	47

Abbreviations: AFB1, aflatoxin B1; CDD, choline‐deficient diet; DEN, diethylnitrosamine; IP, intraperitoneal injection; 2‐AFF, 2‐acetylaminoflourine.

^a^ The pattern was diet‐induced tumor without drug involvement.

### 1.1 Diet‐induced animal models

A choline‐deficient diet (CDD) reproduces the pathological mechanisms underlying the development of non‐alcoholic steatohepatitis (NASH) in humans and its progression to severe stages of HCC.^23^ As an oncogenic factor, CDD can contribute to transforming non‐alcoholic fatty liver disease (NAFLD) into HCC in transgenic mice.^24^ However, it is worth noting that this method requires a long induction time of at least 52 weeks or more.^25^, ^26^ CDD combined with other oncogenic agents constructing HCC animal models could shorten the induction time and improve the success rate. There was a marked elevation in the content of alpha1‐fetoprotein in both the liver and plasma of rats fed with CDD plus DEN, accelerating tumor formation.^27^

High‐fat diet (HFD) is known to be harmful and increases the incidence of HCC. HFD induces hepatic lipid accumulation resulting in lipotoxicity, hepatocyte endoplasmic reticulum stress, inflammation, and hepatocyte injury, leading to spontaneous HCC.^28^ Besides, HFD could promote hepatocarcinogenesis via the accumulation of acylcarnitine and increasing some glucose‐mediated metabolic changes.^29^, ^30^ In addition, cholesterol drives NAFLD‐related HCC through the sequential progression of steatosis, steatohepatitis, fibrosis, and eventually HCC.^31^ Considering the long induction time, combined high‐fat, high‐fructose, and high‐cholesterol diet, with a low weekly dose of intraperitoneal CCl_4_ as an accelerator, a murine NASH model exhibits a rapid progression of advanced fibrosis at 12 weeks and HCC at 24 weeks, and mimics the histological, immunological, and transcriptomic features of human NASH.^32^

A simple induced diet, easy to operate, with low‐cost, is often combined with other carcinogenic agents to induce HCC. Both CDD and HFD could recapitulate the progressive stages of hepatitis, fibrosis, and cancer, closely resembling human disease.^23^, ^32^ Although a previous report has uncovered a dissimilarity between mice and humans, more convincing and accepted evidences show closely similarity of functional pathways of gene expression and immune abnormalities, demonstrating the feasibility of the murine model.^32^, ^33^

### 1.2 DEN‐induced animal models

DEN is a common oncogenic agent that induces DNA damage by alkylating DNA structures or converting cytochrome P450 enzymes in hepatocytes to alkylated metabolites, leading to carcinogenesis.^34^, ^35^ A previous study showed that DEN‐induced gene expression patterns were similar to those in the poor survival group of human HCC.^36^ A single DEN intraperitoneal injection is the classical modeling approach.^8^, ^37^ HCC induced by a single injection occurred after at least 6 months, although the success rate was up to 100%.^38^ The induction of a single DEN injection depends on the administered dose and sex, age, and mouse strain.^39^, ^40^ Younger mice have higher hepatocyte proliferation rates, leading to faster‐developing HCC.^41^ The sex‐related difference in HCC incidence is due to the inhibitory effect of estrogen on hepatocarcinogenesis.^42^ The drawbacks of a single intraperitoneal injection of DEN are the long induction and the inability to mimic the process of fibrosis and cirrhosis during hepatocarcinogenesis.

To overcome the drawbacks of a single injection, long‐term intraperitoneal injection of DEN in rats for 20 weeks combined with oral 0.014% DEN in mice for 15 weeks induced fibrosis and cirrhosis during HCC development.^43^, ^44^ Liver injury induced by DEN is related to drug concentration, and chronic administration of DEN at 20 mg/kg induces HCC in mice, while DEN at 2.5 and 5 mg/kg could be used to elucidate its synergistic effect with other hepatotoxic agents.^45^

In addition, DEN can be combined with other methods to construct HCC models. DEN as a trigger followed by partial hepatectomy, named the Solt–Farber protocol, was originally proposed in 1977 based on the oval cell theory, which can shorten induction time.^46^ In addition, Schiffer's protocol and the modified Solt–Farber protocol combined with 2‐acetylaminoflourine (2‐AFF) can be used in HCC studies.^47^, ^48^ DEN, combined with other oncogenic agents, can mimic the pathophysiological process of human HCC and is a common method for modeling chemical induction. DEN into 2‐week mice, followed by repeated injections of CCl_4_ weekly from 6 weeks, was used to study the mechanism of drug treatment.^49^ In a previous study, alcohol‐DEN mice had more severe hepatocellular damage, advanced fibrosis, and earlier presence of HCC than single‐pair DEN/alcohol‐fed mice.^50^

### 1.3 CCl_4_ ‐induced animal models

CCl_4_ is hepatotoxic due to the formation of reactive oxygen species, contributing to HCC through repeated acute liver injury and inflammation, mimicking the process of hepatitis, fibrosis, cirrhosis, and HCC in humans, which is often applied to study the classical development process.^51^, ^52^

CCl_4_, combined with other methods, is commonly used to construct HCC models considering the long induction time and low success rate of single CCl_4_. At 24 weeks, 10% and 100% of mice developed HCC in the single CCl_4_, and western diet plus CCl_4_ treated groups, respectively.^32^ The combination of CCl_4_ and ethanol can promote CCl_4_ absorption. Mice would develop alcoholic fatty liver disease with significant fibrosis within 7 weeks, similar to the fibrosis, proliferation, and inflammation pattern observed in human disease.^53^ As previously noted, the DEN + CCl_4_ protocol is a common modeling method for studying the mechanisms of hepatocarcinogenesis and drug treatment.^54^ Injection of CCl_4_ followed by injection oncogenic hepatocytes from transgenic mice to establish HCC mice with fibrosis is pathologically similar to the process in humans.^55^

### 1.4 Other drug‐induced animal models

AFB1 is carcinogenic due to DNA mutation of p53, a biomarker of HCC.^56^ Intraperitoneally injection of AFB1 into mice established HCC models after 12 months.^57^ The hepatotoxicity of AFB1 was related to the mouse strain. DBA/2 J mice were threefold more sensitive to AFB1‐induced HCC and acute toxicity than C57BL/6J mice.^58^ 2‐AAF induces the early appearance of progenitor cells and accelerates DEN‐induced hepatocarcinogenesis in rats, suggesting the viability of the DEN + 2‐AAF protocol.^59^, ^60^

## 2 IMPLANTATION ANIMAL MODELS

Implantation models are established by transplanting tumor cells or tissues into animals, which are the most widely used models in current HCC research (Figure 2).^61^ Based on the graft site, they are divided into orthotopic and ectopic models. Implantation models are classified as allograft and xenograft models based on whether the grafts and hosts are from the same or different species.

### 2.1 Allograft animal models

Allograft models are established by transplanting tumor cells or tissues from the same species into hosts by orthotopic or ectopic means (Table 2). These models use homozygous immunocompetent hosts, and are mostly used to study the mechanisms of immune responses and immunotherapeutic efficacy.^62^ Commonly used hepatoma cell lines including BNL‐MEA, Hepa1‐6, BNL, and H22 cells can be used to establish orthotopic or ectopic murine model in immune‐related studies.^63^, ^64^, ^65^ In addition to common mouse HCC cell lines, seeding tumorigenic hepatocytes from Simian vacuolating virus 40 (SV40) T antigen (Tag) transgenic mice into immunocompetent mice is a novel orthotopic mouse model of HCC.^61^

**TABLE 2:** Allograft animal models of hepatocellular carcinoma.

Graft	Cell number	Calculation formula	Host animal	Graft site	Induction time	Tumor size	References
Hepa1‐6	5 × 10^6^	200 μL	BALB/C mice	Unilateral lumbar cord and rib	35 days	8–10 mm in diameter	62
BNL	3 × 10^5^	‐^a^	BALB/c mice	The left liver lobe	7 days	10–20 mm^3^ and 60–100 mm^3^ on Day 7 and Day 14, respectively	63
H22	1 × 10^7^	150 μL	BALB/c mice	The right anterior axilla	‐^a^	100 mm^3^	64
Tag tumorigenic hepatocytes from MTD2 mice	5 × 10^5^ and 5 × 10^6^	‐^a^	C57BL/6 mice	Intravenous (tail vein), subcutaneous, intraperitoneal, and intrasplenic inoculation	4 weeks	130–180 mm^3^	61

^a^ Parameters are not mentioned in articles.

Animal mutations have been described in allograft models using immunocompetent mice and mouse derived HCC cell. However, owing to species differences in the genome, tumor microenvironment, and immune response, whether animal studies can explain the mechanism of tumor occurrence and immune response in humans, remains to be studied further, limiting the practical applications of allograft models.

### 2.2 Xenograft animal models

Xenograft models refer to orthotopic or ectopic transplantation of human tumor cells or tissues into immunodeficient hosts to reduce graft‐versus‐host disease (GVHD) (Table 3) and are widely used in drug screening and mechanism researches.^4^, ^66^ Injecting BEL7404 cells subcutaneously into BALB/c nude mice was common in studying anti‐tumor therapy.^67^ Subcutaneous injection of human hepatoma cells may be beneficial to check therapeutic effects because tumors are easily accessible and can be dynamically monitored for monitoring of tumor growth. However, subcutaneous injections have neglected the microenvironment of the liver and rarely metastasize.

**TABLE 3:** Xenograft animal models of hepatocellular carcinoma.

Graft	Cell number	Calculation formula (μL)	Host animal	Graft site	Induction time	Tumor size	References
Huh7	1 × 10^6^	100	Athymic nude mice	The dorsal flank	‐^a^	200–300 mm^3^	66
HCCLM3	1 × 10^7^	200	Athymic BALB/c nude mice	The right underarm region	6 weeks	525.9 ± 250.8 mm^3^	68
PLC5	1 × 10^6^	100	NCr athymic nude mice	Subcutaneous	‐^a^	60–500 mm^3^	71
Hep3B‐hCG	1 × 10^6^	10	CB17 SCID mice	Liver	‐^a^	‐^a^	9
HepG2	5 × 10^5^	25	BALB/c nude mice	Liver	10 days	‐^a^	69
PLC5	1 × 10^6^	20	NCr athymic nude mice	Liver	1 week	‐^a^	71

^a^ Parameters are not mentioned in articles.

In addition, the dual vascularization of the liver and its special connection to the intestine suggest that it is necessary to establish orthotopic models via intrahepatic, intrasplenic, or intraportal injection, although the surgical procedures are technically demanding.^5^ In orthotopic models, tumors occur in the liver and the surrounding microenvironment, mimicking tumorigenesis, invasion, and metastasis of human HCC. HCCLM3 cells were injected subcutaneously into mice to form tumors, and then the tumors were transplanted into the liver of another nude mouse with a success rate of 90%; this model was confirmed to be reliable by imaging and immunohistochemistry.^68^ And Huh‐7, Hep3B‐hCG, and HepG2 cells establishing orthotopic humanized HCC models were successfully used to study the resistance mechanism, anti‐tumor therapy, and multimodal imaging evaluation for monitoring tumor growth.^9^, ^69^, ^70^ Notably, simultaneously constructed orthotopic and ectopic xenograft mice in the same study would be better to assess the consistent anti‐tumor effect of OSU‐HDAC42.^71^

Transplanting patient‐derived tumor tissue into experimental animals, namely patient‐derived xenograft (PDXs) models, maintains the original characteristics of HCC specimens at the genetic and biological levels, and is often used for drug screening and efficacy research.^72^, ^73^, ^74^ This model has been widely used since it applied to nude mice in 1969.^75^ The study by Fidler indicated that implanting human tumor cells into the organ of nude mice resulted in much higher metastatic rates.^76^ Sun et al. successfully established the LCI‐D20 model (Liver Cancer Institute, passage time‐20 days), an orthotopic PDX model with 100% transplantability and metastatic ability maintained for 18 passages.^77^ Notably, various manifestations in mice are similar to those in patients with HCC, thus, verifying the feasibility of this model.

Owing to grafts derived from humans, xenograft hosts can closely mimic the growth and development process of human tumors. These models provide a simple method to study the effects of novel therapeutic strategies on human HCC cells, avoiding the bias of irrelevant mutations that only occur in mouse HCC tumor cells.^78^ Nevertheless, it should be noted that xenograft models cannot simulate the body's immune response process, given that immunodeficient animals are incapable of generating immune responses and surveillance of tumors.

### 2.3 Humanized immune system animal models

These models refer to animals carrying the human immune system and tumor cells or tissues that simulate the development and immune environment in the human body, with the essential property of the anti‐tumor immune response mimicking that of humans.^79^ Human peripheral blood mononuclear cells or CD34^+^ hematopoietic stem cells were transplanted into immunodeficient hosts; then human tumor cells or tissues were transplanted into hosts, which were applied to study the immunotherapy mechanism.^80^, ^81^ In addition, hepatoma stem cells can be isolated from hepatoma cells or fragments and implanted into immunodeficient animals.^82^ Sub‐renal transplantation of human fetal thymus or fetal liver tissue into mice followed by injection of autologous CD34^+^ hematopoietic stem cells is not suitable for large‐scale application because of source problems and rare models.^83^ The use of these models are limited by high technical requirements, long periods, difficult operation, GVHD, and hypoplasia of CD34^+^ hematopoietic stem cells, etc.^84^

## 3 GEM

**FIGURE 3:**
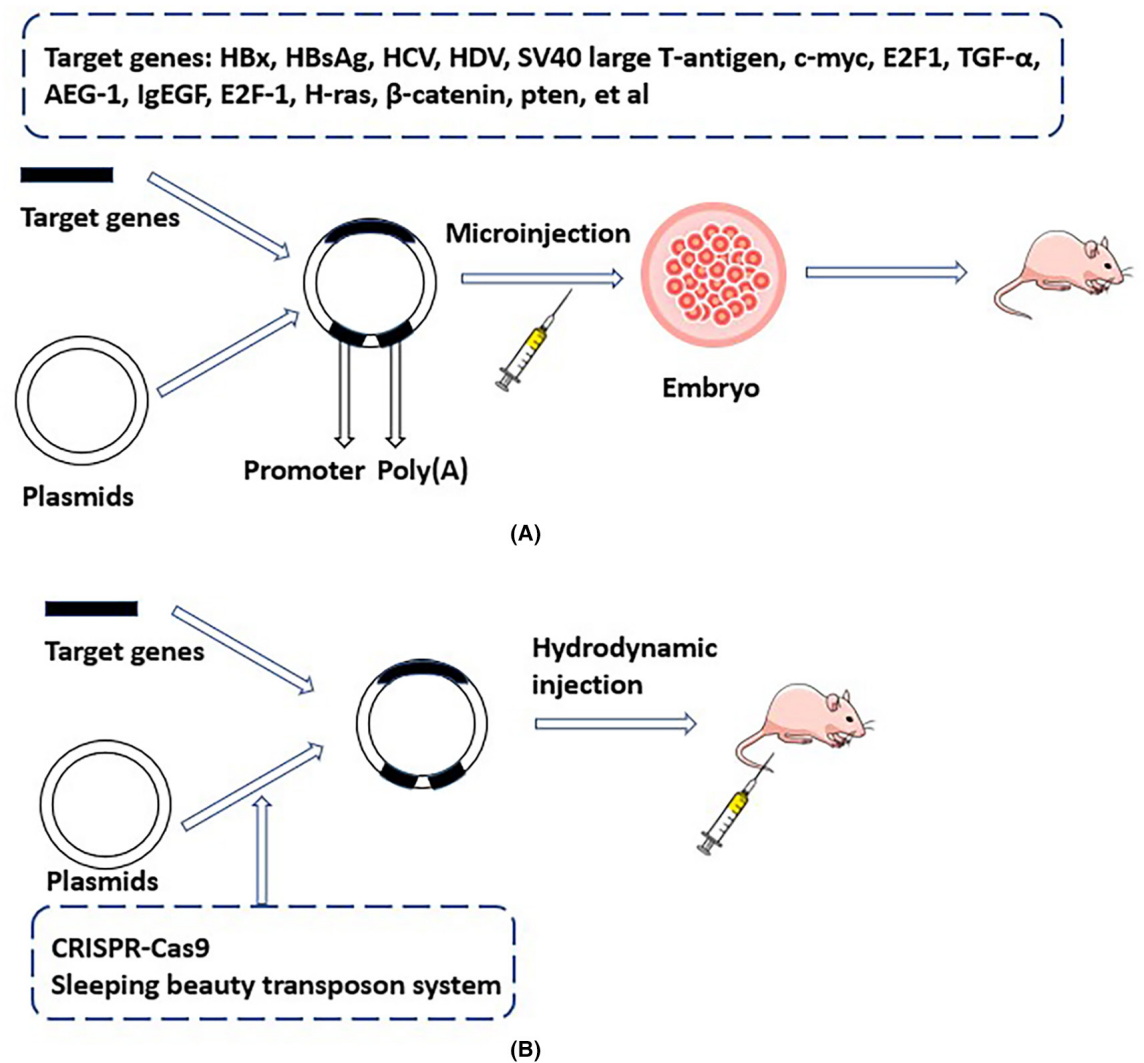
Transgenic mouse model of hepatocellular carcinoma. (A) Traditional transgenic mouse model. (B) Hydrodynamics‐based transfection mouse model with the assistance of CRISPR‐Cas9 and the sleeping beauty transposon system.

HCC is a multistep biological process with recurrent genetic and epigenetic alterations that occur during the progression towards malignant transformation.^85^ Various GEM have been established and described, and the most common way to develop cancer models is to activate oncogenes or inactivate tumor suppressor genes (Figure 3A).^86^, ^87^ GEM of HCC with single or multiple mutations have been widely used, and models with alterations in growth factors or the tumor microenvironment have been reported.^39^, ^88^ Clustered regularly interspaced short palindromic repeats‐associated protein 9 system (CRISPR‐Cas9) directly mutates anti‐oncogenes or oncogenes improving the accuracy of gene editing (Figure 3B).^89^ Moreover, Cre‐LoxP recombination, microRNA delivery, and tetracycline (Tet)‐controlled systems further enable the introduction of liver‐specific mutations, making gene expression more controllable.^90^, ^91^, ^92^ Hydrodynamics‐based transfection is a physical method to help the liver accept exogenous DNA, and the sleeping beauty transposon system stably mediates the chromosomal integration of transposons, representing an attractive gene transfer strategy.^93^, ^94^ And even more surprising, transgenic mouse models harboring specific fragments of hepatitis viral genomes have been developed to study the effect of the human hepatitis virus on HCC.^95^, ^96^ GEM are also widely used in tumor biology studies, investigating the occurrence, progression, and metastasis of tumors, immunity and metabolism, screening drug targets, therapeutic effects, and drug resistance.^7^, ^97^

The hosts of GEM include zebrafish, pigs, and mice. Transgenic zebrafish are experimental tools to study tumor biology and drug treatment. However, unpartitioned liver microstructure, small livers, and lack of a tractable zebrafish embryonic stem cell culture system, limit the clinical application.^17^, ^18^, ^98^ Like humans in anatomy, physiology, and genetics, pigs can also be used to construct the oncopig model.^99^, ^100^ However, pig models have limitations, such as the large size and difficulty of the technical operation. Since the first successful construction in 1974, transgenic mice have been rapidly developed and widely used.^101^, ^102^ Different from the above animal models, mice have the advantages of small body size, easy feeding, strong fecundity and short cycle, which are commonly used as GEM, and this section focuses on transgenic mice (Table 4).

**TABLE 4:** Genetically engineered animal models of hepatocellular carcinoma.

System	Animal strain	Transgene	Promoter	Percentage of tumor	Induction time	References
Viral transgenic models	CD‐1 mice	HBx	HBV	H9 84%; E1 homozygous 43%; E2 heterzygous 6%	H9 16.7 ± 2.5; E1 homozygous 23.0 ± 1.6; E2 heterzygous 24.0	109
	C57BL/6 mice	HBx+p21	HBV	Male: p21^HBx/+^ heterozygotes 60.0% and p21^HBx/HBx^ homozygotes 63.6%; Female: p21^HBx/+^ heterozygotes 53.3% and p21^HBx/HBx^ homozygotes 42.9%	At least 15 months in male and female	111
	C57BL/6 mice	HBsAg+p21	HBV	Male: p21^HBsAg/+^ heterozygotes 53.3% and p21^HBsAg/HBsAg^ homozygotes 72.7%; no female	Male: at least 15 months; no female	111
	C57BL/6 mice	HCV core	HBV	C21: male 25.9%; C49: male 30.8%; no female	Male: at least 15 months; no female	96
	C57BL/6 mice	HCV cDNA	The serum amyloid P component promoter	SC11: 9.1%; SC19: 10%	28–34 months	114
	C57BL/6 mice	SV40 large Tag	Alb	100%	6–9 weeks	119
Conditional transgenic models	Alb/AEG‐1 × Alb/c‐myc mice	AEG‐1, c‐myc	Alb	Male: wild‐type 0%; Alb/AEG‐1 0%; Alb/c‐myc 100%; Alb/AEG‐1/c‐myc 100% (with lung metastases)	Male: Alb/c‐myc 10 months; Alb/AEG‐1/c‐myc 10 months	125
	Alb/c‐myc × Alb/E2F1 mice	c‐myc, E2F1	Alb	C‐myc/E2F1 100%; c‐myc 23%; E2F1 60%	c‐myc/E2F1 6 months; c‐myc 12 months; E2F1 12 months	
	(C57BL/6J × CBA/J) × CD1 mice	c‐myc, TGF‐α	Alb	C‐myc/TGF‐α male 100%, female 30% (aged 8–10 months)	2–4 months	126
	C57BL/6J × CBA/J mice	c‐myc	Alb	C‐myc male 65.2%, female 10% (aged 18–20 months)	10–12 months	126
	CD1 mice	TGF‐α	Metallothionein	Male 63.6%	12 months	130
	Catnb^lox(ex3)^ × Tg^lox(pA)H‐ras^ mice	H‐ras, β‐catenin	Cytomegalovirus	Catnb^lox(ex3)^:Tg^lox(pA)H‐ras*^ (HR‐7) 100%; Tg^lox(pA)H‐ras*^ (HR‐7) 100%; Catnb^lox(ex3)^ 0%; wild‐type 0%	Catnb^lox(ex3)^:Tg^lox(pA)H‐ras*^ (HR‐7) 1–4 weeks; Tg^lox(pA)H‐ras*^ (HR‐7) 6 months	133
	Pten^flox/flox^ × AlbCre mice	Pten	Alb	AlbCrePten^+/+^ 0%; AlbCrePten^flox/+^ 0%; AlbCrePten^flox/flox^ male 83.3%, female 50% (aged 74–78 weeks)	40 weeks	135
MicroRNA transgenic models	LKO: Mir122^loxP^ × albumin‐Cre mice; KO: Mir122^loxP^ × E2a‐Cre mice	miR‐122	Alb	LKO: male 50%, female 10%; KO: male 50%, female 47.4%	LKO: 12–17 months; KO: 11–15 months	137
	B6D2F2 mice	miR‐221	EII‐α1‐antitrypsin	Male 50%, female 0%	9 months	91

Abbreviations: AEG‐1, astrocyte elevated gene‐1; Alb, albumin; E2F1, E2 promoter‐binding factor 1; HBsAg, hepatitis B virus surface antigen; HBV, hepatitis B virus; HBx, hepatitis B virus X protein; HCV, hepatitis C viral; KO, knockout; LKO, liver‐specific knockout; Pten, phosphatase and tensin homolog deleted from chromosome 10; SV40, Simian vacuolating virus; Tag, T antigen; TGF‐α, transforming growth factor‐alpha.

### 3.1 GEM of viral gene

Chronic HBV infection is a common cause of HCC. The HBV genome encodes four viral gene products, of which the HBx and hepatitis B virus surface antigen (HBsAg) are currently the most studied. HBx is a multifunctional regulatory protein that participates in hepatocarcinogenesis by stimulating the expression of c‐myc, activating p53, regulating transcription factors, and driving the G/S cycle via ARRB1‐mediated autophagy.^103^, ^104^, ^105^, ^106^ HBx also induces high levels of MEK–ERK signal activation, facilitating tumor vascular invasion.^107^ Koike et al. constructed HBx transgenic mice and further showed that the sustained expression of the HBx gene may initiate hepatocarcinogenesis by DNA synthesis and hepatocyte transformation.^108^

HBsAg evokes hepatocarcinogenesis through DNA oxidative damage and mutations in the hepatocyte.^109^ In HBV Alb‐1 mice, HBsAg‐induced hepatocellular injury within 4 months progressed to HCC within 12–20 months.^110^ On this basis, the introduction of p53 and phosphatase and tensin homolog deleted from chromosome 10 (Pten) dual mutation into HBV Alb‐1 mice to form HCC mice after 8 months provides a fast and convenient transgenic system with HBV infection characteristics, which is suitable for mechanism and drug screening of HBV‐related HCC.^89^ HBx and HBsAg transgenic mice can be used to study the different roles of HBsAg and HBx in HBV‐related hepatocarcinogenesis.^111^

The hepatitis C virus (HCV) core protein alters the oxidant/antioxidant state in the liver, contributing to the development of HCV‐related HCC.^112^ HCV core gene transgenic mice developed hepatic steatosis at an early stage and HCC at 16 months, verifying that HCV core protein plays a major role in HCV carcinogenesis.^96^ Additionally, HCV transgenic mice could be applied to study the role of iron overload and drug‐induced hepatocyte injury in hepatocarcinogenesis.^113^, ^114^ HDV transgenic mice expressing the functional receptor for HBV and HDV, the human sodium taurocholate cotransporting peptide, have inherited susceptibility to HDV and provided opportunities for studying viral pathogenesis, immune response, and HDV therapeutics.^115^

In addition, genes of SV40, encoding large Tag and small Tag, inactivate the tumor suppressor gene p53 of the host cell, causing carcinogenesis.^116^ SV40 transgenic mice formed tumor nodules 6–9 weeks or later, which are close to the clinical situation and induce the growth of autologous HCC.^117^, ^118^ Based on this model, it can be studied the endothelial transdifferentiation in HCC development and the mechanism of immune tolerance.^118^, ^119^

### 3.2 GEM of conditional gene

Instead of directly transferring exogenous genes, GEM of conditional genes target liver‐specific promoters to drive the expression of oncogenes, such as albumin, metallothionein, transthyretin, and liver activator protein (LAP).^92^, ^120^, ^121^, ^122^, ^123^ C‐myc overexpression in mice using albumin or LAP promoter and transforming growth factor‐alpha (TGF‐α) transgenic mice through the metallothionein promoter can be used to study the pathogenesis and hepatocarcinogenesis.^92^, ^123^, ^124^ C‐myc combined with others in transgenic models shorten tumorigenic time in that c‐myc often interacts with other genes or growth factors participating in HCC development.^125^, ^126^ C‐myc/IgEGF double‐transgenic mice developed HCC at 3–6 months, shorter than single transgenic mice.^127^ C‐myc/TGF‐α and c‐myc/E2 promoter‐binding factor 1 (E2F1) transgenic mice are involved in HCC development, characterized by genomic instability and β‐catenin activation, respectively.^120^ Using c‐myc/p53^−/−^ mice, a study has shown that β‐catenin activation promotes immune escape and resistance to anti‐PD‐1 therapy in HCC.^128^ In addition, the Iqgap2^−/−^ transgenic mouse closely recapitulated many molecular signatures of human HCC, in which dysregulation of Wnt/β‐catenin signaling played an important role.^129^

Alterations in the β‐catenin gene lead to the development of HCC in humans and mice through dysregulation of the Wnt/β‐catenin pathway.^130^, ^131^ Wnt/β‐catenin signaling may cooperate with oxidative stress metabolism and Ras/mitogen‐activated protein kinase pathways in hepatocarcinogenesis.^132^ H‐ras mutation alone rapidly causes hepatocellular dysplasia without autonomous growth, yet HCC develops in all mice with mutated β‐catenin and H‐ras genes, suggesting that β‐catenin mutations play a critical role in hepatocarcinogenesis by cooperating with another oncogene.^133^ Activated AKT and β‐catenin induced microscopic tumor foci by 4‐week in mice, in which synergistic activation of AKT and β‐catenin rapidly induced the progression from adenomas to HCC.^134^ Multiple gene interventions are more likely to induce HCC formation.

Inducible transgenic systems allow temporally controlled genetic changes. Tet‐controlled systems, virus‐mediated Cre‐loxP delivery system and tamoxifen‐regulated Cre‐loxP system are common applied. Crossing TRE‐myc mice with a transgenic line of LAP‐tetracycline transactivator (tTA) to study the effects of myc inactivation, LAP‐tTA/tet‐off myc conditional transgenic mice overexpressing myc in adult mice reproducibly induced HCC, revealing the pluripotent capacity of existing in a state of tumor dormancy.^92^ Besides, in mice with hepatocyte‐specific Pten mutations based on the virus‐mediated Cre‐loxP delivery system, AlbCrePten^flox/flox^ mice developed HCC without hepatocarcinogenesis in AlbCrePten^+/+^ and AlbCrePten^flox/+^ mice.^135^ Moreover, tamoxifen‐regulated Cre‐loxP system is a broadly valuable tool for temporally regulating gene activation/inactivation in mouse embryos, adults, and culture systems.^136^

### 3.3 GEM of microRNA

MicroRNAs (miRNAs) are abnormally expressed in HCC and are related to the degree of HCC differentiation, which provide a promising therapeutic strategy for HCC.^137^ Recently, HCC models have been constructed by dysregulating miRNAs to explore the role of miRNAs in the pathogenesis, metastasis and invasion of HCC. A previous study showed that miR‐122 is the most abundant miRNA in the liver, and its dysregulation is a key factor contributing to the development of HCC.^138^ Based on the Cre‐loxP system, miR‐122a knockout (KO) mice and liver‐specific knockout (LKO) mice were successfully generated, which underwent the process of hepatic steatosis, hepatitis, fibrosis and HCC.^139^, ^140^ Notably, a striking disparity of HCC incidence based on sex in miR‐122a KO mice recapitulates the disease incidence in humans.^140^

Besides, overexpressing miR‐221‐3p significantly enhances the proliferation, migration, and invasion of hepatoma cells, which is an oncogenic miRNA in HCC.^141^, ^142^ In a transgenic mouse model, upregulation of miR‐221 spontaneously resulted in visible neoplastic lesions starting at 9 months of age, which developed a significantly higher number and larger tumor lesions after treatment with DEN.^91^

## 4 COMBINED INDUCTION OF MULTIPLE APPROACHES

The single modeling approach generally has a long modeling period and cannot completely simulate the development of HCC in humans; therefore, multiple modeling methods are frequently combined to establish HCC animal models. In addition to DEN + CCl_4_, HCC mice induced by injecting CCl_4_, followed by oncogenic hepatocytes from transgenic mice, underwent inflammation and fibrosis of the liver.^55^ DEN/phenobarbital induced a significantly higher incidence of HCC in transgenic mice than in wild‐type mice, with marked activation and infiltration of macrophages.^10^ Both single‐injection DEN combined with HFD feeding in transgenic mice, and long‐term administration of CCl_4_ combined with a choline‐deficient l‐amino‐acid‐defined diet in wild‐type mice, formed NASH‐induced HCC, imitating the process of tumor occurrence in human.^143^ C‐myc transgenic mice fed methionine CDD were also used to accelerate hepatocarcinogenesis.^24^

In addition, multiple models were synchronously applied to the same study to verify the reliability of the finding, considering that different models have different cancer‐inducing mechanisms.^71^, ^144^ For example, we could use genetic, orthotopic tumor, chemically induced, and orthotopic allograft models to study the correlation between interleukin‐11 levels and postsurgical HCC recurrence.^145^ We could also use knock‐in, chemically induced, orthotopic, and PDX mice simultaneously to explore the combined inhibition of both homologous recombination and non‐homologous end‐joining as a potential therapy for HCC.^146^

Different kinds of HCC models have their own advantages and disadvantages. On the whole, induced animal models simulate the process of human liver disease with low cost, high success rate, and easy controllability, and are commonly used for HCC pathogenetic mechanism and drug treatment research. Implantation models have small individual differences, increased survival rates, and short induction time, and are convenient for drug screening and objectively evaluating curative effects. Among these, PDXs mimic the tumor microenvironment of human HCC, and humanized immune system models reproduce the complex human immune system and realistic tumor microenvironment that are suitable for immune‐related research. GEM mimic the biological characteristics of human HCC and explain the pathogenesis of HCC at the genetic level for widely various tumor‐related studies with great technical difficulties and costs, which limit the practical application. Combining multiple approaches to construct HCC models, which is increasingly widely used, compensates for the shortcomings of single factors as much as possible.

In conclusion, animal models are basic research tools and are particularly important for studying disease pathogenesis, drug screening, and therapeutic targets. Although none of the current models can completely replicate the real human disease situation, there has been great progress in the research and construction of HCC animal models. When making selections, researchers should comprehensively consider many factors including research purpose, modeling period, feeding environment, host strain, and funding. In addition, multiple methods can be combined to construct HCC models. Particularly, multiple models are recommended to use in the same study and the experimental findings can be synchronously verified in diverse models. In the future, researchers should combine advanced techniques of DNA manipulation with implanted murine or human cell lines to simulate human HCC in animal models.

## AUTHOR CONTRIBUTIONS

**Jing Li:** Conceptualization (equal); writing – original draft (equal). **Xin Wang:** Writing – review and editing (equal). **Mudan Ren:** Data curation (equal). **Shuixiang He:** Conceptualization (equal); writing – review and editing (equal). **Yan Zhao:** Conceptualization (equal); writing – review and editing (equal).

## FUNDING INFORMATION

This work was supported by the Natural Science Foundation of Shaanxi Province (Program No. 2022JM‐456).

## CONFLICT OF INTEREST STATEMENT

All authors have no financial or personal conflict of interest to declare. All authors have approved the final statement and the content of the manuscript.

## Data Availability

N/A.
